# Association between vitamin B2 intake and prostate-specific antigen in American men: 2003–2010 National Health and Nutrition Examination Survey

**DOI:** 10.1186/s12889-024-18582-y

**Published:** 2024-05-03

**Authors:** Jia-jie Lv, Lin-jie Zhang, Xiang-Meng Kong, Yan Zhao, Xin-yu Li, Jing-bing Wang, Xi-tao Yang, Zhi-hua Cheng, Wen-zhi Li, Xu-hui Wang, Cheng-hao Yang

**Affiliations:** 1 Department of Vascular Surgery, Shanghai Putuo People’s Hospital, School of Medicine, Tongji University, Huangpu District, No.1291 Jiangning Road, Shanghai, 200060 China; 2 Department of Vascular Surgery, Shanghai Ninth People’s Hospital, Shanghai JiaoTong University School of Medicine, Huangpu District, No.639 Zhizaoju Road, Shanghai, 200011 PR China; 3 Department of Cardiology, Shanghai Ninth People,s Hospital, Shanghai Jiao Tong University School of Medicine, Huangpu District, No.639 Zhizaoju Road, Shanghai, 200011 China; 4 Department of Nursing, Shanghai Ninth People’s Hospital, Shanghai Jiao Tong University, Huangpu District, No.639 Zhizaoju Road, Shanghai, 200011 PR China; 5 Department of Plastic and Reconstructive Surgery, Shanghai Ninth People’s HospitalShanghai Jiao Tong University School of Medicine, Huangpu District, No.639 Zhizaoju Road, Shanghai, 200011 China; 6 Department of Interventional Therapy, Multidisciplinary Team of Vascular Anomalies, Shanghai Ninth People’s Hospital, Shanghai Jiao Tong University, Huangpu District, No.639 Zhizaoju Road, Shanghai, 200011 PR China; 7 Department of Neurosurgery, Shanghai Ninth People’s Hospital, Shanghai Jiao Tong University School of Medicine, Huangpu District, No.639 Zhizaoju Road, Shanghai, 200011 China; 8 Department of Urology, Shanghai General Hospital, Shanghai Jiao Tong University School of Medicine, Shanghai, 200080 China

**Keywords:** Prostate-specific antigen, Vitamin B2, Prostate cancer, National Health and Nutrition Examination Survey (NHANES)

## Abstract

**Background:**

Accumulating evidence suggests a pivotal role of vitamin B2 in the pathogenesis and progression of prostate cancer (PCa). Vitamin B2 intake has been postulated to modulate the screening rate for PCa by altering the concentration of prostate-specific antigen(PSA). However, the relationship between vitamin B2 and PSA remains indeterminate. Hence, we conducted a comprehensive evaluation of the association between vitamin B2 intake and PSA levels, utilizing data from the National Health and Nutrition Examination Survey (NHANES) database.

**Methods:**

From a pool of 20,371 participants in the NHANES survey conducted between 2003 and 2010, a cohort of 2,323 participants was selected for the present study. The male participants were classified into four distinct groups based on their levels of vitamin B2 intake. We employed a multiple linear regression model and a non-parametric regression method to investigate the relationship between vitamin B2 and PSA levels.

**Results:**

The study cohort comprised of 2,323 participants with a mean age of 54.95 years (± 11.73). Our findings revealed a statistically significant inverse correlation between vitamin B2 intake (mg) and PSA levels, with a reduction of 0.13 ng/ml PSA concentration for every unit increase in vitamin B2 intake. Furthermore, we employed a fully adjusted model to construct a smooth curve to explore the possible linear relationship between vitamin B2 intake and PSA concentration.

**Conclusions:**

Our study in American men has unveiled a notable inverse association between vitamin B2 intake and PSA levels, potentially posing a challenge for the identification of asymptomatic prostate cancer. Specifically, our findings suggest that individuals with higher vitamin B2 intake may be at a greater risk of being diagnosed with advanced prostate cancer in the future, possibly indicating a detection bias. These results may offer a novel explanation for the observed positive correlation between vitamin B2 intake and prostate cancer.

**Supplementary Information:**

The online version contains supplementary material available at 10.1186/s12889-024-18582-y.

## Introduction

Prostate cancer is one of the most widespread cancers among men globally, with an estimated lifetime risk of 1 in 8 men [1, 2]. Early diagnosis is pivotal for successful treatment, as prostate cancer generally progresses slowly and exhibits no symptoms until it has metastasized to other regions of the body [3]. Hence, screening tests are employed to detect prostate cancer at an early stage, before the onset of symptoms. The prostate-specific antigen (PSA) test is a prevalent screening test for prostate cancer, which measures the concentration of PSA, a protein produced by the prostate gland, in the blood [4]. In the presence of prostate cancer, the PSA level may become elevated [5]. Therefore, the PSA test is instrumental in detecting elevated PSA levels in the blood, indicating the potential presence of prostate cancer.

Nonetheless, the PSA test's use as a screening tool for prostate cancer remains contentious since itmay provide inaccurate diagnoses. Elevated PSA levels may also occur due to conditions other than cancer, such as benign prostatic hyperplasia (BPH) or prostate inflammation [6–8]. Furthermore, certain nutritional supplements, including folic acid, calcium, and multivitamins [9, 10], have been shown to alter PSA levels and affect cancer detection that can increase the likelihood of missed prostate cancer screening. The consumption of excessive doses of nutritional supplements may result in health hazards and toxicity, and some dietary nutritional supplements have been linked to a higher risk of PCa in smokers [11, 12]. Hence, it is imperative to examine the relationship between various nutrient elements and PCa and PSA levels. An increasing number of studies have highlighted the association between vitamin B2 and prostate cancer pathogenesis, albeit with conflicting explanations [13–15]. Some studies have demonstrated that vitamin B2 may contribute to the development and progression of prostate cancer [16, 17]. Nevertheless, some studies have posited vitamin B2 as a protective factor, with its derivative riboflavin functioning as an androgen antagonist [18].

We thus posit a plausible link between vitamin B2 and PSA metabolism, which could potentially result in detection bias in prostate cancer diagnosis. Remarkably, this phenomenon has yet to be reported in previous literature. Our working hypothesis is that vitamin B2 intake may modulate PSA concentrations in the US male population, subsequently impacting prostate cancer screening outcomes and prognosis. To investigate this hypothesis, we conducted a secondary data analysis of the US National Health and Nutrition Examination Survey (NHANES), controlling for a multitude of confounding factors. Our objective was to investigate the relationship between vitamin B2 intake and PSA concentrations in US men while reconcilingthe discordant findings.

## Materials and methods

### Data availability

The National Health and Nutrition Examination Survey (NHANES), spearheaded by the National Centers for Disease Control (CDC) and Prevention National Health Statistics Center, has been underway since 1960 to assess the health and nutritional status of individuals in the United States. The NHANES website (www.cdc.gov/nchs/nhanes, accessed on 7 October 2021) provides comprehensive information about the survey's population and methodology. NHANES protocols have obtained approval from the Health Statistics research ethics review board of the National Center, adhering to stringent ethical guidelines.

### Study population

**Fig. 1:**
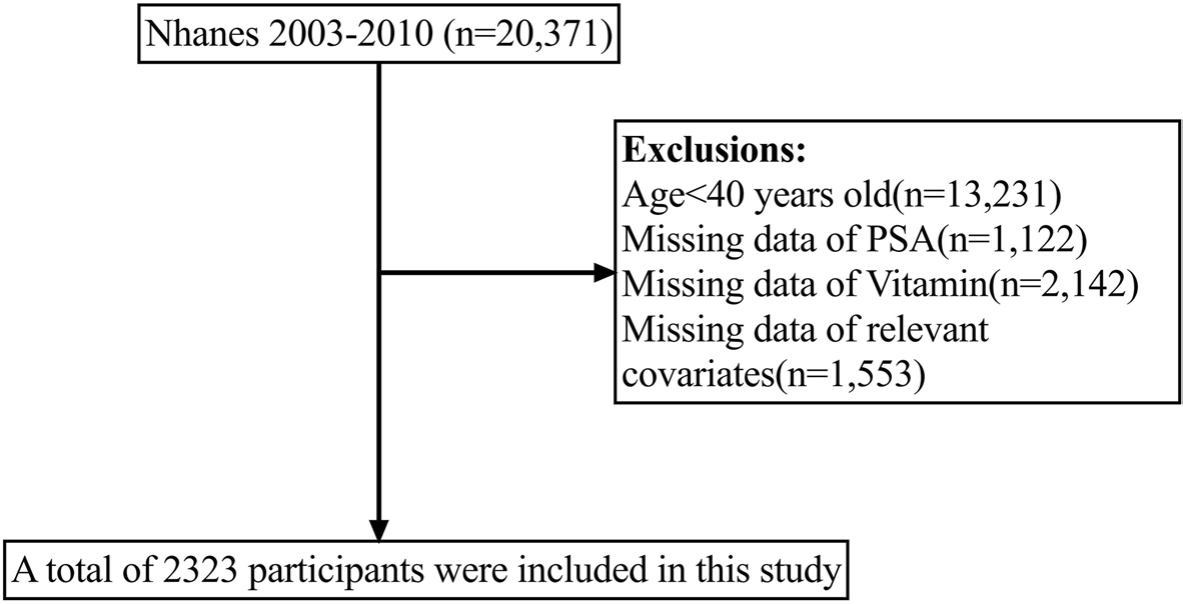
Flow chart for participants recruitment of this study, NHANES 2003–2010

Our study encompassed four NHANES components conducted between 2003 and 2010 (i.e., 2003–2004, 2005–2006, 2007–2008, and 2009–2010). The dataset incorporated PSA concentrations, vitamin intake, sociodemographic data, laboratory data, medical examinations, and personal life history, among other relevant data. Participants who failed to meet the following exclusion criteria were excluded from our study: (1) age < 40 years (*n* = 13,231); (2) lack of PSA(1,122); (3) lack of vitamin intake (*n* = 2,142); and (4) missing other relevant data (*n* = 1,553). Consequently, 2,323 of the 41,156 participants were included in our study (Fig. 1). Our study followed the World Medical Association Declaration of Helsinki guidelines. NHANES served as the basis for our data analysis.

Vitamins intake measurement Dietary vitamin intake data were obtained from the NHANES Dietary Interview-Total Nutrient Intake Data file. Nutrition data in this file were collected through a 5-step USDA automated multipass method (AMPM) combined with the USDA Dietary Research Food and Nutrition Database [19, 20]. Supplemental vitamin intake was obtained from the NHANES Dietary Supplements section household interview, which collects information on dietary supplement use. To improve accuracy, participants showing supplement containers answered questions about duration of use, frequency over the past 30 days, and daily frequency. For this study, supplement users were persons taking vitamins in any form. Average daily supplemental vitamin intake was calculated by dividing total intake over 30 days by 30. Total daily vitamin intake combined supplemental and dietary intake. For individuals with no supplementation or missing data, total intake equaled dietary intake.

### PSA measurement

Total PSA concentrations were detected using the Hybritech PSA immunoassay method on the Beckman Access system, which automatically measures light production of reacted samples. Free PSA concentrations were measured by the Access Hybritech two-site immunoenzymatic sandwich assay. The PSA ratio was calculated by dividing free PSA by total PSA and multiplying by 100. The total PSA cutoff value to dichotomize was 4.0 ng/mL, and the PSA ratio cutoff was 15% [21].

### Covariates

In our regression analyses, we evaluated potential associations with confounding variables [22, 23], including age, race/ethnicity, marital status, smoking history, body mass index (BMI), metabolism (MET), education, poverty-to-income ratio, triglycerides, serum total cholesterol, high-density lipoprotein, low-density lipoprotein, and related diseases. The registered population's race was classified as non-Hispanic white, non-Hispanic black, Hispanic (Mexican Americans and other Hispanic Americans), and other. Smoking status was determined as previous (history of smoking), never (never smoked), and current (new smoker). Poverty-to-income ratios were computed based on poverty guidelines for household size to reflect the participants' economic and social standing.

### Statistical analysis

Following CDC guidelines (https://www.cdc.gov/nchs/nhanes/index.htm, accessed on October 7, 2021), we utilized vitamin B2 and PSA levels for statistical analysis. We gave careful consideration to masking variance, taking into account the sample weights for each participant in the NHANES and applying recommended weighting methods. The data are expressed as mean ± standard deviation or proportions, as appropriate. Weighted t-tests were employed for continuous variables, while weighted χ2 tests were utilized for categorical variables to compare differences between different vitamin B2 intake groups (quartile). Participants in this study had PSA measurements ranging from 0.07 to 64.21 ng/ml. To ensure that PSA was normally distributed, we logarithmically processed the PSA values (Figure S1). The excluded population was compared with the study population at baseline to reduce sampling error (Table S1). Our objective was to examine the potential relationship between vitamin B2 intake and PSA concentrations in specific participants. Firstly, we classified the continuous variable of vitamin B2 intake into quartile concentrations. Categorical variables were computed using weighted chi-square, while continuous variables were determined using weighted linear regression models between quartiles. Secondly, we analyzed the effect of different vitamin intake on PSA by employing restricted spline models to evaluate the trend between different vitamin types and PSA and constructing three weighted univariate and multiple linear regression models. Crude Model, minimally adjusted Model (Model 1), and fully adjusted model (Model 2) were utilized to assess vitamin intake and calculate the linear relationship between vitamin intake and PSA concentration. In addition, subgroup analyses were conducted using stratified multiple linear regression analysis to ascertain the stratified association between vitamin B2 and PSA. Finally, we used the penalty spline method to develop a fully adjusted model and perform a smooth curve fit of the model to explore the potential linear relationship between vitamin B2 and PSA. To prevent bias due to missing data, we collated the NHANES database, and all analyses were performed using the statistical packages R and SPSS. A P value less than 0.05 was regarded as statistically significant.

### Modeling approach

To assess the association between vitamin B2 intake and PSA levels, we used several modeling approaches: First, vitamin B2 intake was divided into quartile to allow comparisons across levels of intake. Weighted t-tests and chi-square tests were used to compare participant characteristics across tertiles. Second, we constructed linear regression models with vitamin B2 intake as a continuous or categorical (quartile) predictor and log-transformed PSA as an outcome. Crude and adjusted models were used to control for confounding factors. Then, stratified analyses were performed according to demographic and clinical factors to explore the association between vitamin B2 intake and PSA levels. Finally, we generated a penalized spline model to explore the potential linear relationship between continuous intake of vitamin B2 and PSA concentrations after adjusting for covariates. In summary, the modeling approach quantified a linear predictive relationship between vitamin B2 intake and PSA levels while controlling for potentially confounding variables. This allowed us to estimate the PSA change per 1-unit increase in vitamin B2 intake.

## Results

### Basic characteristics of participants

We collected data on vitamin B2 intake and serum PSA from 41,156 NHANES participants 40 years of age or older, leaving 2323 participants in the study after the exclusion of relevant missing data. Table 1 provides an overview of the basic demographic characteristics and other covariates of the enrolled participants, according to quartile of vitamin B2 intake, according to National Health and Wellness Survey data from 2003 through 2010. The mean age of the study population was 54.95(± 11.73) years, and the median PSA concentration was 0.96 ng/ml. There were significant differences in age, race, marital status, education, PIR, smoking status and PSA concentration among different vitamin B2 groups (all *P* < 0.05, Table 1).

**Table 1:** Characteristics of participants by Vitamin B2, NHANES 2003–2010

**Variable**	**Total**	Tertiles of vitamin B2 levels, mg^c^			*P*
		**Q1**	**Q2**	**Q3**	
**Age(years, SD**^**a**^**)**	54.95 ± 11.73	56.38 ± 11.95	55.06 ± 11.67	52.68 ± 10.7	< 0.0001
**Race/ethnicity (n, %)**					< 0.0001
Mexican American	374(16.1)	190(8.83)	115(4.57)	69(4.95)	
Non-Hispanic Black	388(16.7)	223(12.81)	116( 6.32)	49( 4.35)	
Non-Hispanic White	1239(53.34)	410(64.79)	538(82.81)	291(85.17)	
Other Hispanic	222(9.56)	113(4.72)	71(2.71)	38(2.85)	
Other race/ethnicity	100(4.3)	65(8.85)	25(3.59)	10(2.68)	
**Marital status (n, %)**					0.01
Living with partner	123(5.29)	57(4.75)	34(2.61)	32(7.32)	
Married	1952(84.03)	824(85.17)	749(89.49)	379(83.65)	
Single	248(10.68)	120(10.09)	82( 7.90)	46( 9.03)	
**Education (n, %)**					**0.002**
High school	857(36.89)	372(35.22)	324(33.36)	161(34.58)	
Less than high school	312(13.43)	187(9.71)	75(3.98)	50(3.84)	
More than high school	1154(49.68)	442(55.07)	466(62.65)	246(61.58)	
**PIR**^**b**^** (SD)**	3.48 ± 1.65	3.14 ± 1.61	3.73 ± 1.64	3.54 ± 1.65	< 0.0001
**BMI** **(kg/m**^**2**^**, SD)**	28.81 ± 5.42	28.87 ± 5.5	28.70 ± 5.3	28.91 ± 5.45	0.81
**MET (SD)**	5304.83 ± 6962.64	4729.35 ± 6552.28	5227.33 ± 6687.48	6271.92 ± 8125.67	0.02
**TG (mmol/L, IQR)**	1.32(0.94)	1.28(0.89)	1.31(0.97)	1.41(0.89)	0.39
**TC (mmol/L, IQR)**	5.07(1.42)	5.02(1.45)	5.04(1.50)	5.15(1.32)	0.08
**HDL (mmol/L, IQR)**	1.19(0.44)	1.19(0.44)	1.19(0.46)	1.19(0.47)	0.48
**LDL (mmol/L, IQR)**	3.08(1.19)	3.07(1.22)	3.05(1.18)	3.16(1.09)	0.15
**Smoking status (n, %)**					0.01
Former	873(37.58)	387(36.14)	333(36.14)	153(32.79)	
Never	991(42.66)	431(47.71)	373(48.73)	187(42.38)	
Now	459(19.76)	183(16.16)	159(15.13)	117(24.84)	
**PSA (ng/ml, IQR)**	0.96(1.19)	1.00 (1.3)	0.98(1.21)	0.86(0.92)	< 0.001

^a^*SD* Standard Deviation, *IQR* Interquartile Range

^b^*PIR* Poverty to income ratio, *BMI* Body mass index, *TG* triglyceride, *TC* total cholesterol, *HDL* high-density lipoprotein, *LDL* low-density lipoprotein, *PSA* Prostate specific antigen

^c^Vitamin B2 quartile ranges: Quartile 1 = 0.03 to 2.03, Quartile 2 = 2.03 to 3.20, Quartile 3 = 3.20 to 26.52

### Linear regression analysis of vitamin intake and PSA

**Fig. 2:**
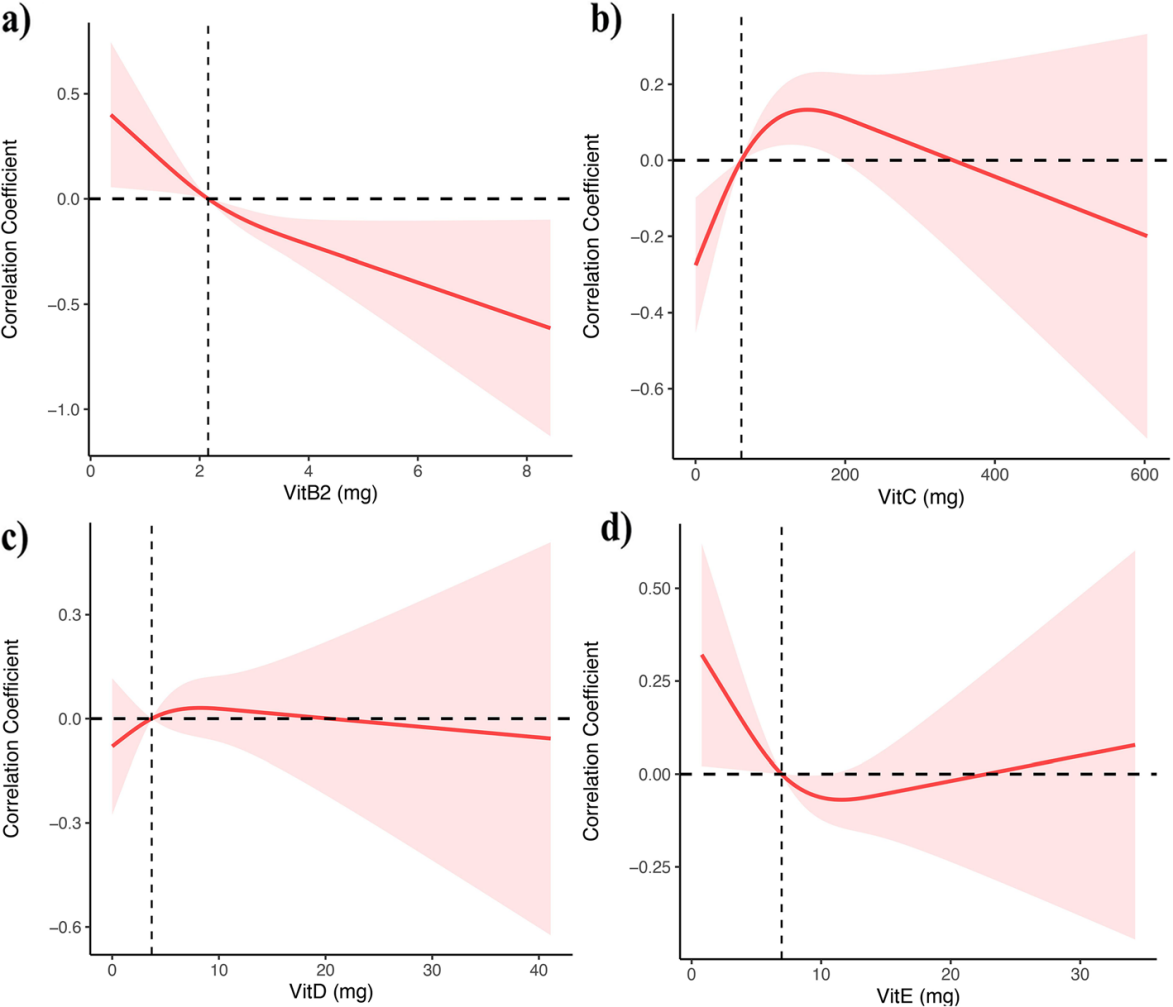
Correlation coefficient between NHANES PSA index and VitB2, VitC, VitD and VitE during 2003–2010. Adjusted for age, sex, race, education, marital status, income, BMI, smoking and alcohol consumption.The shaded area represents the 95% confidence interval

Figure 2 illustrates the relationship between the intake of four vitamins and PSA through a constrained cubic spline model analysis. After adjusting for age, race, education, marital status, poverty-to-income ratio, body mass index, smoking status, total cholesterol, triglycerides, high-density lipoprotein, and low-density lipoprotein, we found that vitamin B2 intake was negatively correlated with PSA (*P* < 0.01). However, no association was observed between the intake of other vitamins and PSA (Table 2). Participants were grouped according to the tertile or interquartile range of each vitamin, and the lowest concentration group (Q1) was used as the reference for the analyses. In the crude model, multiple linear regression analysis showed that vitamin B2 in Q4 group was negatively correlated with PSA (OR = -0.10, 95%CI: -0.26–0.06). The association remained significant in model 1 with adjustment for age and race (OR = -0.01, 95%CI: -0.15 to 0.14). In model 2, after adjusting for education level, marital status, poverty to income ratio, body mass index, smoking status, total cholesterol, triglyceride, high-density lipoprotein and low-density lipoprotein, the association still existed (OR = -0.04, 95%CI: -0.02 to 0.13). In contrast, no association was found between the intake of other vitamins and PSA (Table 2).

**Table 2:** Univariate and multivariate analyses by the weighted linear model

	Crude Model^a^			Model 1^b^			Model 2^c^		
Exposure^d^	OR	95%CI	*P*	OR	95%CI	*P*	OR	95%CI	*P*
Vitamin A									
Continuous	0.01	0,0.01	0.85	0	0,0	0.89	0	0,0	0.92
Q1	ref			ref			ref		
Q2	0.08	-0.05,0.20	0.21	0.09	-0.02,0.20	0.08	0.09	-0.02,0.20	0.09
Q3	0.11	-0.01,0.23	0.08	0.11	0,0.21	0.14	0.10	0.03,0.21	0.04
Q4	0.05	-0.09,0.19	0.48	0.04	-0.07,0.16	0.34	0.06	-0.06,0.16	0.44
P for trend	0.49			0.56			0.58		
Vitamin B1									
Continuous	-0.05	-0.11,0.01	0.1	-0.01	-0.07,0.05	0.78	-0.01	-0.07,0.05	0.76
Q1	ref			ref			ref		
Q2	0.01	-0.13,0.15	0.86	0.02	-0.11,0.15	0.76	0.02	-0.11,0.15	0.74
Q3	-0.02	-0.13,0.09	0.70	0.03	-0.07,0.14	0.52	0.04	-0.08,0.15	0.51
Q4	-0.08	-0.24,0.08	0.31	0.01	-0.15,0.17	0.93	0.01	-0.15,0.16	0.92
P for trend	0.23			0.92			0.91		
Vitamin B2									
Continuous	-0.05	-0.08,-0.02	0.003	-0.05	-0.07,-0.02	0.002	-0.02	-0.04,0.01	0.02
Q1	ref			ref			ref		
Q2	-0.01	-0.11,0.10	0.92	0.03	-0.08,0.13	0.60	0.05	-0.05,0.16	0.65
Q3	0.02	-0.12,0.15	0.80	-0.06	-0.06,0.17	0.03	0.05	-0.08,0.17	0.44
Q4	-0.10	-0.26,0.06	0.04	-0.01	-0.15,0.14	0.04	-0.04	-0.02,0.13	0.02
P for trend	0.05			0.04			0.04		
Vitamin B6									
Continuous	-0.04	-0.06, -0.02	0.01	-0.02	-0.04, 0.01	0.15	-0.02	-0.04,0.01	0.15
Q1	ref			ref			ref		
Q2	0.08	-0.02,0.19	0.12	0.12	0.03,0.21	0.01	0.12	-0.02,0.22	0.02
Q3	0.05	-0.05,0.15	0.29	0.10	0.02,0.19	0.02	0.11	-0.02,0.19	0.02
Q4	-0.06	-0.18,0.06	0.34	0.04	-0.06,0.14	0.43	0.04	-0.06,0.14	0.41
P for trend	0.22			0.68			0.65		
Vitamin B12									
Continuous	-0.01	-0.01, 0	0.04	0	-0.01, 0	0.33	0	-0.01, 0	0.35
Q1	ref			ref			ref		
Q2	0.03	-0.08,0.14	0.60	0.03	-0.08,0.14	0.60	0.03	-0.09,0.14	0.63
Q3	0.03	-0.10,0.16	0.62	0.06	-0.06,0.18	0.29	0.06	-0.06,0.19	0.30
Q4	-0.09	-0.02,0.01	0.07	-0.03	-0.12,0.07	0.55	-0.03	-0.12,0.07	0.56
P for trend	0.09			0.62			0.65		
Vitamin C									
Continuous	0	0,0	0.20	0	0,0.01	0.15	0.02	0.01,0.04	0.14
Q1	ref			ref			ref		
Q2	0.07	-0.03,0.18	0.17	0.03	-0.07,0.14	0.53	0.05	-0.09,0.20	0.51
Q3	0.20	0.10,0.31	< 0.001	0.13	-0.03,0.23	0.01	0.13	0.03,0.24	0.01
Q4	0.20	0.08,0.32	0.002	0.16	-0.06,0.26	0.07	0.17	0.07,0.27	0.06
P for trend	< 0.001			0.002			0.45		
Vitamin D									
Continuous	0	-0.01,0.01	0.55	0	0,0.01	0.40	0.01	0,0.02	0.41
Q1	ref			ref			ref		
Q2	0.08	-0.07,0.24	0.29	0.06	-0.09,0.20	0.43	0.05	-0.09,0.20	0.45
Q3	0.05	-0.05,0.15	0.33	0.03	-0.06,0.11	0.51	0.02	-0.06,0.11	0.59
Q4	0.05	-0.01,0.19	0.51	0.06	-0.07,0.18	0.39	0.06	-0.08,0.19	0.39
P for trend	0.65			0.49			0.50		
Vitamin E									
Continuous	-0.02	-0.04,0.03	0.96	-0.03	-0.09,0.04	0.08	-0.02	-0.08,0.03	0.08
Q1	ref			ref			ref		
Q2	-0.09	-0.24,0.06	0.21	-0.05	-0.17,0.07	0.41	-0.05	-0.18,0.07	0.39
Q3	-0.07	-0.21,0.07	0.32	-0.01	-0.14,0.12	0.88	-0.01	-0.14,0.12	0.90
Q4	-0.03	-0.15,0.08	0.54	0.04	-0.05,0.14	0.35	0.04	-0.05,0.14	0.33
P for trend	0.86			0.20			0.16		
Vitamin K									
Continuous	-0.02	-0.10,0.07	0.50	0.01	-0.08,0.14	0.53	-0.01	-0.02,0.01	0.50
Q1	ref			ref			ref		
Q2	-0.03	-0.17,0.11	0.68	0	-0.15,0.15	0.98	0	-0.15,0.15	0.99
Q3	0.02	-0.10,0.14	0.76	0.06	-0.06,0.17	0.31	0.06	-0.05,0.17	0.27
Q4	-0.04	-0.17,0.08	0.48	-0.02	-0.13,0.09	0.68	-0.02	-0.13,0.09	0.93
P for trend	0.63			0.87			0.90		

^a^Crude Model: no covariates were adjusted

^b^Model 1: adjusted for age; race

^c^Model 2: adjusted for age; race; poverty: income ratio; education level; marital status; BMI; smoking; HDL; LDL; TG; TC

^d^Vitamin A quartile ranges: Quartile 1 = 0 to 299, Quartile 2 = 299 to 538, Quartile 3 = 538 to 845, Quartile 4 = 845 to 12413; Vitamin B1 quartile ranges: Quartile 1 = 0.03 to 1.16, Quartile 2 = 1.16 to 1.64, Quartile 3 = 1.64 to 2.22, Quartile 4 = 2.22 to 8.68; Vitamin B2 quartile ranges: Quartile 1 = 0.14 to 1.53, Quartile 2 = 1.53 to 2.16, Quartile 3 = 2.16 to 2.94, Quartile 4 = 2.94 to 26.52; Vitamin B6 quartile ranges: Quartile 1 = 0.03 to 1.41, Quartile 2 = 1.41 to 1.97, Quartile 3 = 1.97 to 2.78, Quartile 4 = 2.78 to 38.54; Vitamin B12 quartile ranges: Quartile 1 = 0 to 2.76, Quartile 2 = 2.76 to 4.71, Quartile 3 = 4.71 to 7.36, Quartile 4 = 7.36 to 131.56; Vitamin C quartile ranges: Quartile 1 = 0 to 25.25, Quartile 2 = 25.25 to 61.30, Quartile 3 = 61.30 to 127.40, Quartile 4 = 127.40 to 1617.80; Vitamin D quartile ranges: Quartile 1 = 0 to 1.70, Quartile 2 = 1.70 to 3.70, Quartile 3 = 3.70 to 6.70, Quartile 4 = 6.70 to 79.30; Vitamin E quartile ranges: Quartile 1 = 0.08 to 4.53, Quartile 2 = 4.53 to 6.95, Quartile 3 = 6.95 to 10.34, Quartile 4 = 10.34 to 54.09; Vitamin K quartile ranges: Quartile 1 = 0.2 to 37.3, Quartile 2 = 37.3 to 62.9, Quartile 3 = 62.9 to 113.2, Quartile 4 = 113.2 to 2146.7

### Subgroup analysis

Table 3 displays the results of the subgroup analysis of the relationship between vitamin B2 intake and PSA. Stratified analyses were conducted by age, race, marital status, education, body mass index (BMI), and household income ratio. The results of subgroup analyses are shown in Table 3. Subgroup analysis showed that all p values for interaction between subgroups were > 0.05, suggesting no significant effect change. In conclusion, the effect of vitamin B2 on PSA remained consistent across different subgroups, including gender, age, race, MET, PIR, BMI, TG, TC, HDL, LDL, and smoking status.

**Table 3:** Subgroup analysis of the association between vitamin B2 and PSA

Character	HR(95% CI)	*P*	*P* for interaction
**Age**			0.535
[40,56]	-0.02(-0.045,0.005)	0.108	
(56,80]	-0.04(-0.106,0.026)	0.222	
**Race**			0.067
Mexican American	-0.016(-0.085,0.054)	0.64	
Non-Hispanic White	-0.042(-0.073,-0.011)	0.01	
Other Hispanic	-0.098(-0.218,0.021)	0.102	
Non-Hispanic Black	-0.044(-0.124,0.036)	0.265	
Other race/ethnicity	-0.223(-0.401,-0.044)	0.019	
**Marital status**			0.118
Married	-0.056(-0.089,-0.022)	0.002	
Single	0.006(-0.059,0.072)	0.845	
Living with partner	-0.013(-0.083,0.057)	0.702	
**Education status**			0.441
Less than high school	-0.037(-0.121,0.047)	0.372	
High school	-0.03(-0.067,0.008)	0.114	
More than high school	-0.062(-0.111,-0.012)	0.016	
**Smoking status**			0.442
Never	-0.051(-0.092,-0.010)	0.017	
Former	-0.065(-0.140,0.010)	0.087	
Now	-0.025(-0.048,-0.001)	0.044	
**BMI**			0.528
[14.2,25.0)	-0.07(-0.141,0.000)	0.05	
[25.0,30.0)	-0.048(-0.084,-0.013)	0.009	
[30.0,65.4]	-0.023(-0.084,0.038)	0.446	
**PIR**			0.736
(0,1.3]	-0.028(-0.072,0.015)	0.192	
(1.3,]	-0.058(-0.101,-0.014)	0.011	
(3.5,5]	-0.051(-0.118,0.016)	0.131	
**MET**			0.155
[40,1320]	-0.047(-0.153,0.060)	0.379	
(1320,4800]	-0.089(-0.135,-0.042)	< 0.001	
(4800,55440]	-0.014(-0.039,0.011)	0.269	
**TG**			0.079
[0.2,1.7)	-0.015(-0.065,0.035)	0.549	
[1.7,31.0]	-0.09(-0.154,-0.025)	0.008	
**TC**			0.095
[2.4,5.2)	-0.068(-0.109,-0.026)	0.002	
[5.2,13.7]	-0.024(-0.063,0.016)	0.227	
**HDL**			0.286
[0.44, 1.0]	-0.058(-0.101,-0.016)	0.009	
(1.0,1.55]	-0.034(-0.071,0.004)	0.075	
(1.55,3.72]	-0.078(-0.136,-0.019)	0.011	
**LDL**			0.375
[0.8,3.4]	-0.055(-0.122,0.012)	0.104	
(3.4,7.5]	-0.018(-0.064,0.029)	0.447	

*PIR* Poverty to income ratio, *BMI* Body mass index, *TG* triglyceride, *TC* total cholesterol, *HDL* high-density lipoprotein, *LDL* low-density lipoprotein, *PSA* Prostate specific antigen

### Penalty spline method analysis

**Fig. 3:**
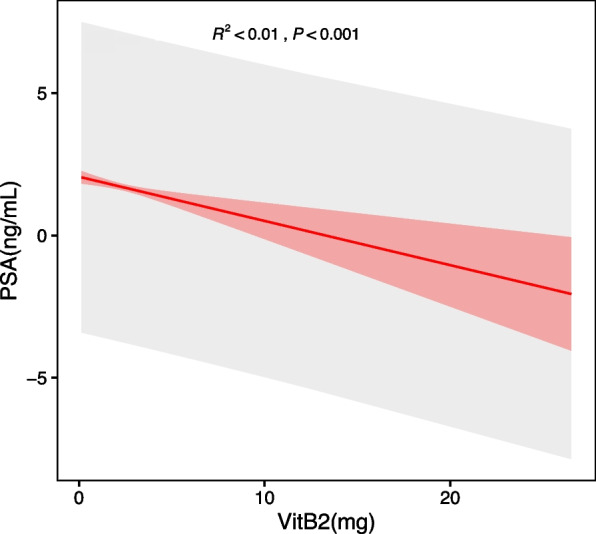
The relationship between Vitamin B2 and prostate-specific antigen (PSA) connections. The shaded area represents the 95% confidence interval

Sensitivity analyses were performed to confirm the accuracy and stability of the results. First, vitamin B2 intake was converted into categorical variables intertiles, as described above, and P values were calculated for trend analysis. Surprisingly, the results for categorical variables were consistent with the effect of riboflavin as a continuous variable (Table 2). To investigate the possible linear relationship between vitamin B2 and PSA concentration, a smooth curve was constructed using the penalized spline method based on the fully adjusted model. According to the fully adjusted model, the relationship between serum triglycerides and PSA concentration was linear after adjustment for other covariates (Fig. 3). The results showed that PSA concentration decreased by 0.13 ng/mL for each 1 mg increase in vitamin B2 intake. These results suggest an inverse correlation between vitamin B2 and PSA concentration.

## Discussion

Our study represents one of the most extensive cross-sectional investigations to date exploring the potential association between vitamin B2 intake and prostate-specific antigen (PSA) levels in men in the United States, based on the NHANES database. Notably, no prior epidemiological study has reported an association between vitamin B2 and PSA levels. Our findings yield two critical insights. Firstly, our analysis reveals a noteworthy association between vitamin B2 intake and PSA levels, even after adjusting for various potential confounding factors. Specifically, serum PSA concentrations tend to decrease to a certain extent with an increase in vitamin B2 intake. Secondly, our results demonstrate that serum PSA concentration decreases by 0.13 ng/ml for each unit (mg) increase in vitamin B2, and this difference is statistically significant. Notably, we did not observe any significant correlation between the intake of other vitamins and serum PSA concentrations. Our findings provide valuable guidance for further investigating the relationship between vitamin B2 and PSA levels. Future studies can focus on identifying the optimal dose or mechanism of vitamin B2 action on PSA levels.

Several prospective studies have previously suggested a potential role for vitamin B2 in the development and progression of prostate cancer [13, 15, 24, 25]. These studies indicate that increasing vitamin B2 intake may be associated with the development of prostate cancer. The mechanisms underlying this association can be summarized as follows: First, flavin mononucleotide (FMN), a derivative of vitamin B2, exhibits anti-androgen activity by inhibiting DHT-induced activity in 22RV1 and LNCAP prostate cancer cells in a concentration-dependent manner. Second, vitamin B2 serves as a cofactor in the methylenetetrahydrofolate reductase reaction, participates in methyl transfer reaction and REDOX reaction, and regulates cell metabolism and gene expression. Third, vitamin B2 plays a role in DNA repair processes, facilitating the repair of damaged DNA and reducing DNA mutations and genetic abnormalities, thereby lowering the risk of cancer [18, 26–28]. Although some studies have reported a relationship between vitamin B2 and the incidence and mortality of prostate cancer, the conclusions are somewhat inconsistent [13–15]. For example, Mattias et al. [13] suggest that vitamin B2 is a risk factor for prostate cancer, and that elevated circulating choline and vitamin B2 concentrations may be associated with an increased risk of prostate cancer. In contrast, another study suggests that vitamin B2 is a protective factor [18], and its derivative FMN may inhibit the binding of DHT to the androgen receptor, serving as a competitive androgen receptor antagonist. Julie et al. [16], in a systematic review, suggested that the conflicting evidence in the literature may be due to the slow progression of prostate tumors and the fact that tumors occur years before diagnosis, and that the direction of the relative risk associated with B vitamin intake may depend on the time between measured intake and diagnosis.

We also hypothesized that vitamin B2 might affect PSA concentration, potentially leading to a bias in detection and a conflict of interpretation. The precise mechanisms underlying vitamin B2-induced PSA decline remain undefined and warrant exploration. As the biologically active form of vitamin B2, flavin mononucleotide (FMN) can interact with steroid receptors involved in prostate carcinogenesis including the androgen receptor (AR) [18]. By binding to AR and blocking dihydrotestosterone-mediated activation, FMN may suppress AR-stimulated PSA production in prostate cells [18]. Additionally, vitamin B2 influences epigenetic mechanisms implicated in prostate cancer development. As an essential cofactor for methyltransferases, vitamin B2 availability regulates gene promoter methylation and expression of tumor suppressors like glutathione S-transferase pi 1 (GSTP1) that are silenced epigenetically during prostate carcinogenesis [29]. Vitamin B2 deficiency may promote prostate cancer progression through aberrant promoter methylation. Furthermore, the role of vitamin B2 as an essential cofactor and regulator of one-carbon metabolism provides another potential explanation [26]. Through modulating methylation patterns and redox status, vitamin B2 could alter gene transcription and metabolic pathways implicated in prostate carcinogenesis and impact PSA generation [16]. Altered vitamin B2 levels may dysregulate mitochondrial function and energy metabolism in prostate tumors, influencing proliferative capacities [30]. Vitamin B2 availability also regulates DNA repair processes that rectify cancer-instigating genetic aberrations [27]. Additionally, the antioxidant properties of vitamin B2 may mitigate oxidative damage linked to prostate cancer development [28]. Elucidating the relative contributions of these putative mechanisms requires further in vitro and in vivo experimentation.

Our findings suggest an inverse association between vitamin B2 and PSA levels, which could result in detection bias and have implications for prostate cancer screening. Specifically, given that vitamin B2 may reduce PSA concentrations in men without prostate cancer, the sensitivity of PSA testing for detecting prostate cancer in men with high vitamin B2 intake may be diminished. Therefore, if vitamin B2 intake reduces PSA production from prostate cancer or modifies the ability of tumor-derived PSA to enter the serum, it may be necessary to adjust the PSA threshold to improve the sensitivity of PSA testing. Further investigations are required to elucidate the mechanisms by which vitamin B2 affects PSA levels and its impact on prostate cancer screening. Moreover, leveraging the PSA-lowering attributes of vitamin B2 by developing vitamin B2-conjugated platforms for targeted PSA suppression specifically within tumor sites could limit side effects compared to traditional anti-androgen therapies. Additional research exploring therapeutic applications is warranted. Furthermore, extrapolating our findings to other malignancies could unveil analogous detection biases perpetuated by vitamin B2 status, presenting avenues for enhancing early diagnosis and treatment of further cancer types. Evaluating markers such as the cancer antigen 125 for ovarian cancer across population subgroups with different vitamin exposures may prove illuminating.

The primary strength of our study lies in the use of a representative, well-documented cohort that integrates expertise in nutrition and clinical medicine to investigate the association between serum PSA concentrations and vitamin intake. Additionally, we employed a restricted cubic spline model and multiple hierarchical linear regression analyses to evaluate the effects of different vitamin intakes on PSA levels. We also performed a sensitivity analysis to assess the impact of potential confounders, and a smooth curve was constructed based on the fully adjusted model to explore the possible linear relationship between vitamin B2 intake and serum PSA concentrations. Furthermore, we weighted the data to ensure that our findings were generalizable to the broader US population. However, our study has some limitations that must be acknowledged. Firstly, dietary fluctuations may not be fully captured by a single 24-h dietary review, making it challenging to infer the long-term effects of vitamin B2 on serum PSA. Secondly, because we used data from cross-sectional surveys, the causal relationship between vitamin B2 and serum PSA remains uncertain. Finally, although we adjusted for several potential confounders, the influence of other factors cannot be completely excluded. While our findings have some medical plausibility, caution must be exercised when interpreting them, and longitudinal studies or clinical trials are needed to validate our results. Moreover, although we adjusted for several potential confounders, the influence of other factors cannot be entirely ruled out. As such, further large-scale, multi-center clinical studies are necessary to confirm the association between vitamin B2 intake and serum PSA concentration and explore its mechanism and clinical applicability.

In summary, our study demonstrates an inverse link between vitamin B2 intake and PSA levels in American men, with potential detection bias ramifications for prostate cancer screening protocols. These results advocate investigations into modifying cut-offs for positive PSA tests among regular vitamin B2 consumers to facilitate early identification of lesions masked by low PSA. Elucidating the molecular underpinnings and long-term impacts of vitamin B2 exposure along with leveraging attributes of vitamin B2 for diagnostic or therapeutic purposes constitutes promising directions for future work.

## Conclusions

In summary, our study offers a novel investigation of the association between vitamin B2 intake and serum PSA concentrations while elucidating the linear shape of this relationship. Our findings demonstrate that vitamin B2 intake is negatively correlated with PSA levels, with serum PSA decreasing as vitamin B2 intake increases. This observation suggests that individuals with higher vitamin B2 intake may be at an increased risk of being diagnosed with advanced prostate cancer in the future, and this detection bias may provide new insights into explaining the positive association between vitamin B2 and prostate cancer.

### Supplementary Information

**Supplementary Material 1.**

## Data Availability

The datasets generated and/or analysed during the current study are available in the Nhanes repository, https://www.cdc.gov/nchs/nhanes/index.htm.
