# Supplementary material for: Association between vitamin B2 intake and prostate-specific antigen in American men: 2003–2010 National Health and Nutrition Examination Survey
Source: BMC Public Health. 2024 May 3;24:1224. doi: 10.1186/s12889-024-18582-y (PMC11067116; doi:10.1186/s12889-024-18582-y)
Supplement: Supplementary file 1 — Supplementary Material 1. [file 12889_2024_18582_MOESM1_ESM.docx]

**Supplementary Material**

**Table S1. Exclusion and characteristics of the study participants, NHANES 2003–2010.** a. SD: Standard deviation; **IQR: Interquartile Range**. b. PIR: **Poverty to income ratio; BMI: Body mass index; TG:** **triglyceride; TC:** **total cholesterol; HDL:** **high-density lipoprotein; LDL:** **low-density lipoprotein; DM: Diabetes mellitus; CKD: Chronic kidney disease; CHD: Coronary heart disease; PSA: Prostate specific antigen.**

****Figure S1. Histogram and Quantile-Quantile Plot of Log-PSA.****

Table S1. Exclusion and characteristics of the study participants, NHANES 2003–2010.

| **Variable** | Research data (n=2,323) | Exclusion of data (n=11,603) | **P** |
| --- | --- | --- | --- |
| **Age (years, SD)** | 60.71±12.95 | 62.81±13.62 | 2.20 |
| **Race/ethnicity (n, %)** |  |  | **0.22** |
| Mexican American | 626(26.95) | 2040(17.58) |  |
| Non-Hispanic Black | 267(11.49) | 2476(21.34) |  |
| Non-Hispanic White | 1162(50.02) | 5795(49.94) |  |
| Other race/ethnicity | 268(11.54) | 1292(11.14) |  |
| **Marital status (n, %)** |  |  | 0.41 |
| Living with partner | 123(5.29) | 477(4.12) |  |
| Married | 1952(84.03) | 9819(84.62) |  |
| Single | 248(10.68) | 1307(11.26) |  |
| **Education (n, %)** |  |  | **0.75** |
| High school | 981(42.23) | 4697(40.48) |  |
| Less than high school | 359(15.45) | 2534(21.84) |  |
| More than high school | 983(42.32) | 4372(37.68) |  |
| **PIR (SD)** | 2.74±1.61 | 2.57±1.57 | 1.22 |
| **BMI (kg/㎡, SD)** | 28.73±5.64 | 28.38±5.84 | 0.24 |
| **MET** **(SD)** | 3388.22±5801.95 | 2860.20±5810.59 | 1.25 |
| **Vitamin A (mcg, SD)** | 649.17±801.96 | 641.92±859.61 | 0.64 |
| **Vitamin B1 (mg, SD)** | 1.73±0.92 | 1.67±0.91 | 0.15 |
| **Vitamin B2 (mg, SD)** | 2.34±1.31 | 2.28±1.25 | 0.66 |
| **Vitamin B6 (mg, SD)** | 2.12±1.36 | 1.99±1.19 | 4.30 |
| **Vitamin B12 (mcg, SD)** | 5.85±8.50 | 5.65±8.95 | 0.21 |
| **Vitamin C (mg, SD)** | 89.44±98.91 | 86.23±93.71 | 0.07 |
| **Vitamin D (mcg, SD)** | 5.09±5.92 | 4.87±6.00 | 0.22 |
| **Vitamin E (mg, SD)** | 7.53±5.41 | 7.01±5.19 | 1.59 |
| **Vitamin K (mcg, SD)** | 100.70±162.22 | 96.72±166.53 | 0.19 |
| **TG (mmol/L,IQR)** | 1.32(0.94) | 1.75(1.44) | 0.74 |
| **TC (mmol/L,IQR)** | 5.07(1.42) | 5.06(1.16) | **0.04** |
| **HDL (mmol/L,IQR)** | 1.19(0.44) | 1.26(0.37) | 0.48 |
| **LDL (mmol/L,IQR)** | 3.08(1.19) | 3.02(0.99) | 0.46 |
| **PSA (ng/ml,IQR)** | 0.96(1.19) | 1.53(2.62) | 0.10 |
| **Smoking status (n, %)** |  |  | 0.32 |
| Former | 895(38.53) | 4597(39.62) |  |
| Never | 981(42.23) | 4200(36.20) |  |
| Now | 447(19.24) | 2806(24.18) |  |

a. SD: Standard deviation; **IQR: Interquartile Range**.

b. PIR: **Poverty to income ratio; BMI: Body mass index; TG:** **triglyceride; TC:** **total cholesterol; HDL:** **high-density lipoprotein; LDL:** **low-density lipoprotein; DM: Diabetes mellitus; CKD: Chronic kidney disease; CHD: Coronary heart disease; PSA: Prostate specific antigen.**


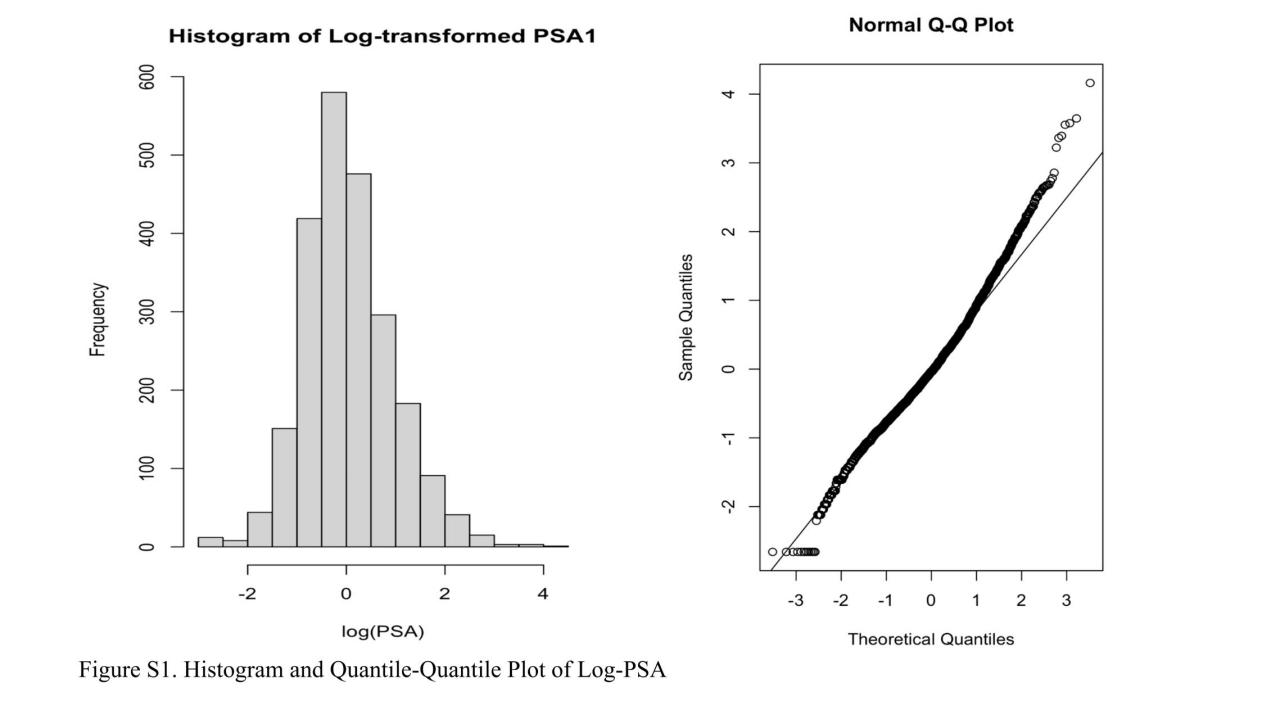


Figure S1. Histogram and Quantile-Quantile Plot of Log-PSA.
